# Factors associated with microbiological contamination of chicken meat marketed in El Salvador

**DOI:** 10.17843/rpmesp.2023.401.12100

**Published:** 2023-03-27

**Authors:** Alejandro López, Tatiana Burgos, Marcela Vanegas, Zaida Álvarez, Yudy Mendez, Edgar Quinteros

**Affiliations:** ¹ National Institute of Health of El Salvador, San Salvador, El Salvador. National Institute of Health of El Salvador San Salvador El Salvador; ² Faculty of Chemistry and Pharmacy, Universidad de El Salvador, San Salvador, El Salvador. Faculty of Chemistry and Pharmacy Universidad de El Salvador San Salvador El Salvador

**Keywords:** Food, Chickens, Salmonella, Escherichia coli, Staphylococcus aureus

## Abstract

**Objective.:**

To determine the hygienic-sanitary factors associated with the microbiological contamination of chicken meat sold at the municipal markets of El Salvador.

**Materials and methods.:**

An analytical cross-sectional study was conducted in 33 municipal markets of the 14 departmental capitals of El Salvador. The sample consisted of 256 out of 456 possible market stalls. A sample of chicken meat was obtained from each market stall. The microbiological analysis was conducted at the National Public Health Laboratory. Frequencies, percentages, measures of central tendency and association were calculated with SPSS version 21.

**Results.:**

Escherichia coli was found in 74% of the samples, Staphylococcus aureus in 24% and Salmonella spp. in 1%. The presence of Salmonella spp. was associated with not using hand sanitizer and not using towels for drying the hands. S. aureus was associated with the use of personal accessories and improper storage. The presence of S. aureus was associated with the lack of hand washing, not using a towel to dry the hands and not wearing an apron.

**Conclusion.:**

The hygienic-sanitary conditions of the handlers and the market stalls were associated with microbiological contamination of chicken meat marketed in El Salvador.

## INTRODUCTION

The microbiological contamination of food is a risk to public health, as it may be the cause of foodborne diseases (FBD) ^1^. Diarrhea is the most frequent acute symptom of FBD, however, these conditions can have severe consequences such as renal failure, liver failure, brain and neural disorders, reactive arthritis, cancer and death ^2^. Flaws during the food production process, inadequate handling and unsanitary conditions are the main factors that influence contamination. Children, the elderly and pregnant women are the most vulnerable groups to FBD ^1^. According to the World Health Organization (WHO), FBD cause around 420,000 deaths worldwide each year and affect one in ten people who consume contaminated food ^3^. The Epidemiological Surveillance System of El Salvador (VIGEPES) ^4^ reported 1735 cases of food poisoning and 1,482,613 cases of diarrhea and gastroenteritis between 2016 and 2020.

*Salmonella* spp., *Escherichia coli* (*E. coli*) and *Staphylococcus aureus* (*S. aureus*) are some of the microorganisms that cause FBD ^3^. *Salmonella* spp. is a bacterium that normally lives in the intestines of many animals, particularly poultry, cattle, swine, reptiles, and others ^5^. *Salmonella* spp. causes the disease known as salmonellosis ^1^^,^^6^.

*E. coli* is a bacterium that lives in the intestines of humans and warm-blooded animals ^7^. The identification of this bacterium in water or food can be associated with fecal contamination. Some pathogenic strains secrete toxins such as Shiga ^8^, and cause severe diseases ^9^. On the other hand, *S. aureus* is a bacterium that mainly inhabits the skin and nasal cavities of humans ^6^; it can also infect food and produce gastroenteritis and, depending on the amount of Colony Forming Units (CFU), it can produce heat-resistant enterotoxins ^10^.

Animal source foods are the most commonly associated with FBD ^11^ and are the most consumed by the population ^3^. For example, chicken meat is an important source of protein and high-quality nutrients ^12^. The main source of microbiological contamination of chicken meat is their microbiota. Contamination can occur due to poor manufacturing practices and flaws in the different stages of production and handling ^13^.

In El Salvador, one of the most important sources of animal protein is chicken meat, due to its availability and low cost ^14^. Between 2018 and 2019, El Salvador produced more than 280 million kilos of chicken meat ^15^. In the country, the main places where chicken meat is sold are the supermarkets and municipal markets. In 2015, a study of 43 supermarkets in the capital of El Salvador reported the prevalence of *Salmonella* spp. (56%), *E. coli* (14%) and *S. aureus* (13%) ^16^; however, there are no similar studies on municipal markets, which are the main places where chicken meat is sold, due to the economic accessibility of this products ^17^. The sanitary conditions of these places are deficient, which could contribute to the high prevalence of these microorganisms; therefore, this study aimed to determine the hygienic-sanitary factors associated with contamination by *Salmonella* spp., *E*. *coli* and *S. aureus* in chicken meat marketed in municipal markets in the departmental capitals of El Salvador.

**KEY MESSAGES**

Motivation for the study. The presence of pathogenic microorganisms in food is a risk to public health. In addition, there is little information in El Salvador about microbiological contamination of food from municipal markets.

Main findings. Three species of microorganisms were identified in chicken meat; their presence was associated with some hygienic-sanitary conditions of handlers and stalls.

Implications. Identifying the factors associated with microbiological contamination in chicken samples can contribute to the creation of public policies aimed at strengthening preventive measures and disseminating good food handling practices.

## MATERIALS AND METHODS

We carried out an analytical cross-sectional study that included samples of raw chicken meat marketed in stalls at the 33 municipal markets of the departmental capitals of El Salvador, from August to November 2017.

**Figure 1:**
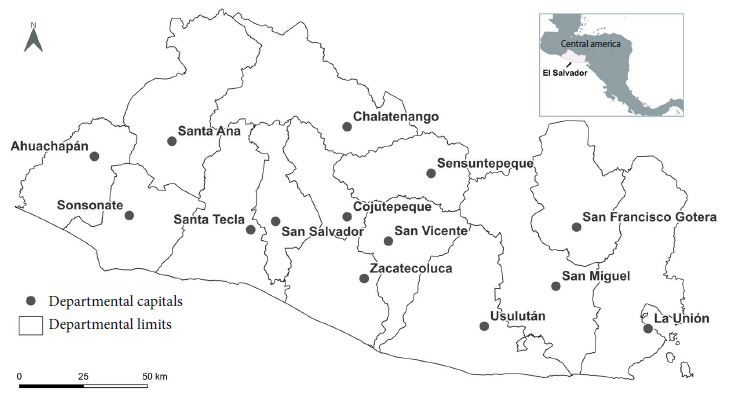
Location of the departmental capitals showing the markets included in the study

A sample of raw chicken meat being sold in stalls at the markets from the 14 departmental capitals of El Salvador was calculated in order to determine the number of samples to include in this study. We conducted a cartographic survey of the stalls with the support of environmental sanitation inspectors from the Ministry of Health and identified 456 stalls. Then, a sketch map of each market was drawn up, in which we located the stalls that sold chicken meat. We identified zones according to the type of commercialization and the internal and external reference points (Figure 1). A correlative number was assigned to each stall and the name of the owner or tenant and the stall number assigned by the market administration were registered.

The sample was calculated by using a formula for finite populations ^18^, with a variance of p=0.56 and q=0.44, a statistical significance of 1.96 and a margin of error of 5%. We obtained a sample size of 222 stalls. The sample was selected by applying the simple random method in Microsoft Excel 2016. A sample of chicken meat was obtained from each stall; each sample consisted of two pieces of raw chicken that were on sale at the counter. All the collected pieces consisted of skin and meat.

Researchers from the Instituto Nacional de Salud of El Salvador (INS) and environmental sanitation personnel from the Ministry of Health of El Salvador collected the sample. The person responsible for collecting the sample explained the importance of the study to the persons in charge of the stalls and requested written informed consent from each of them. Subsequently, data was collected in a form that included information regarding the time and date of collection, stall number and name of the market, code, type of sample, temperature at the time of collection, identification of the stall manager or owner, and the person in charge of collecting the sample. Another form gathered information about the stall, including the use of masks, hand washing, use of personal accessories, makeup, use of aprons and gloves, hand and skin lesions, drainage, presence of vectors outside and inside the stall, presence of garbage outside and inside the stall, presence of stagnant water outside and inside the stall, and presence of domestic animals.

Temperature was measured before collecting the sample by using a food thermometer (COMARK DT400) with a measuring range of -20°C to 100°C. The thermometer was disinfected before each measurement. Each piece of chicken was individually packed in a hermetically sealed Whirl Pak-type plastic bag and identified with an adhesive label including the sample code, the required laboratory analysis, time, date and place of sampling. The methodology established by CODEX Alimentarius ^19^ and the guidelines for sample collection and shipment of the National Public Health Laboratory (LNSP) were used during sample collection and transport. Samples were stored and transported to the LNSP in coolers at a temperature of 4°C. The LNSP sample collection, shipping and receiving form was filled out for each sample.

Samples were analyzed at the LNSP. *Salmonella* spp. was isolated and identified by using the PCR Screening Assurance GDS technique, official method AOAC 2009.03 ^20^. Samples that were initially positive were confirmed by streaking on selective-differential agars. One colony with typical *Salmonella* spp. appearance was isolated from each of the selective-differential media. The isolated strains were identified with the API 20E and VITEK 2 biochemical systems. This methodology only allows determining the presence or absence in 25g of sample.

*E. coli* was quantified in Petrifilm plates using the AOAC 991.14 official method ^21^. The inoculated plates were incubated at 35°C ± 1°C for 48 hours. Blue or bluish-red colonies associated with the formation of gas bubbles were counted when reading the plates. The number of colonies obtained was multiplied by the corresponding dilution factor. *E. coli* results are expressed in colony forming units per gram (CFU/g).

*S. aureus* was quantified in Petrifilm plates using the official AOAC 2003.11 ^22^ method. The inoculated plates were incubated at 35°C ± 1°C for 24 hours. Red-violet colonies were counted when reading the plates. Suspected *S. aureus* colonies were confirmed by placing a Petrifilm Staph Express disk (DNase test) on the plates with distinctive growth. These samples were incubated at 35°C ± 1°C for 3 hours. After incubation, red-violet colonies with a pink zone around the colony were counted. Confirmed isolates (DNase positive) from Petrifilm plates were identified by coagulase test. A positive result indicated the presence of *S. aureus*. The number of confirmed colonies was multiplied by the dilution factor. *S. aureus* results are expressed as CFU/g.

We created a database in Epi Info version 7 with the information collected in the stall inspection form, the good food handling practices inspection form and the results obtained from the laboratory analysis. The database was then exported to Statistical Package for the Social Sciences (SPSS) version 21 for frequency analysis, and calculating the percentage of positive samples and measures of central tendency. Association was assessed by using odds ratio (OR) with a 95% confidence interval (CI).

The study was approved by the National Health Research Ethics Committee in the approval act N° 19/2017, in order to comply with the principles of ethics in health research. All persons, owners of the stalls agreed to participate in the study and signed an informed consent. This document specified the general data of the researchers, the institution, the aim of the study, as well as the selection and sampling procedures. In addition, it was stated that participation was completely voluntary and confidential. Each participant was informed that the samples might be sold or donated.

## RESULTS

**Figure 2:**
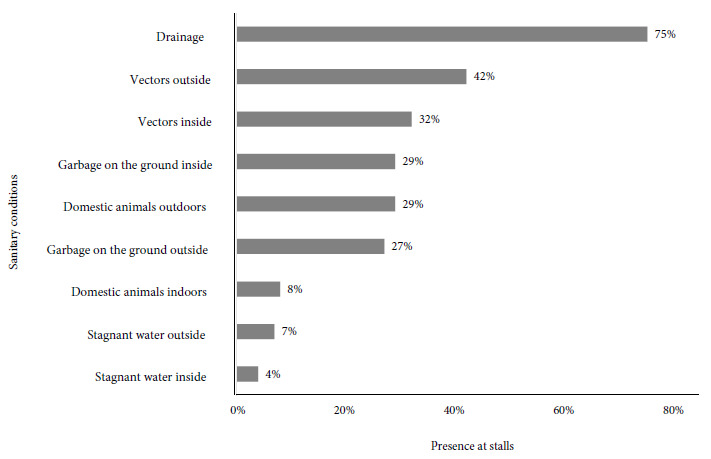
Main sanitary conditions inside and outside stalls selling chicken meat

We assessed the hygienic and sanitary conditions of 221 stalls that sold raw chicken meat. Most of the stalls were located in the departmental capital of San Salvador (24%), followed by San Vicente (9.5%), Santa Ana (9.5%), Sonsonate (8.5%) and, to a lesser extent, La Unión (2.3%). Seventy-five percent of the stalls had drainage, 42% had vectors inside and 32% had vectors outside. In 29% of the stalls, garbage was found inside and in 27% outside. Domestic animals were found outside of 29% of the stalls (Figure 2). The average temperature of the chicken meat was 11.3°C (SD: 8.6°C); The storage temperature of 76.5% of the samples was inadequate (> 4°C).

Regarding the hygienic and sanitary conditions of the handlers, 97.3% did not use gloves, 83.7% did not use aprons and 48.9% washed their hands. Most of the handlers did not have type of hand or skin lesion (Table 1).

**Table 1:** Main hygienic conditions of chicken meat handlers (N=221).

Hygienic conditions of the handler	Yes	No
	n (%)	n (%)
Use of hairnets	51 (23.1)	170 (76.9)
Use of apron	36 (16.3)	185 (83.7)
Use of gloves	6 (2.7)	215 (97.3)
Use of personal accessories	92 (41.6)	129 (58.4)
Use of makeup	37 (16.7)	184 (83.3)
Having long nails	22 (10.0)	199 (90.0)
Hand washing	108 (48.9)	113 (51.1)
Use of hand disinfectant	18 (8.1)	203 (91.9)
Use of paper towels for hand drying after disinfection	2 (0.9)	219 (99.1)
Use of a clean towel for hand drying after hand disinfection	24 (10.9)	197 (89.1)
Hand washing	107 (48.6)	113 (51.4)
Use of soap for hand washing	133 (60.2)	88 (39.8)
Use of clean water for hand washing	139 (62.9)	82 (37.1)
Use of a clean towel to dry hands after hand washing	82 (37.4)	137 (62.6)

Sixteen percent of the samples were positive for *Salmonella* spp. This result is outside the range established by the Central American Technical Regulation RTCA 67.04.50:17 ^23^. Seventy-four percent of the samples showed contamination by *E. coli*, of which 43% were within the acceptable range, and 57% exceeded the standard range established by RTCA 67.04.50:17 (23) (Table 2). Twenty-four percent of the samples were contaminated by *S. aureus*, of which 85% were within an acceptable range and 15% exceeded the range established by Nicaraguan Standard NTON 03-023-12 (24) (Table 2).

**Table 2:** Presence of Salmonella spp., E. coli, and S. aureus in chicken meat compared to international microbiological criteria.

Microorganism	N° of positive samples	Established limits	N° of samples per range	Percentage (%)	Microbiological criteria
*Salmonella* spp.	35	Presence	35	16	Absence at 25g^a^
		Absence	186	84	
*Escherichia coli*	164	<100 CFU/g	96	43	(m) 1x10^2^ CFU/g^a^
		>100 CFU/g	125	57	
*Staphylococcus aureus*	52	<100 CFU/g	188	85	(m) 1x10^2^ CFU/g^b^
		>500 CFU/g	33	15	

a Central American Technical Regulation RTCA 67.04.50:1722

b Nicaraguan Standard NTON 03-023-1223

(m): Microbiological criterion below which the food does not represent a risk to health.

Table 3 shows the variables related to the microbiological contamination of chicken meat. The presence of *Salmonella* spp. was strongly associated with not using hand sanitizer (OR: 3.97; 95%CI: 1.42-11.12). Similarly, not using a clean towel to dry the hands after washing was also associated with the presence of *Salmonella* spp.

**Table 3:** Variables related to the presence of *Salmonella* spp., *E. coli* and *S. aureus* in fresh chicken meat.

Variable	***Salmonella* spp.**		*E. coli*		*S. aureus*	
	OR	95%CI	OR	95%CI	OR	95%CI
Hygienic conditions of the handler						
Did not use hairnets	1.67	0.75‒3.70	0.43	0.22-0.84	1.00	0.47-2.09
Did not wear an apron	0.62	0.20‒1.88	0.47	0.22-1.00	2.46	1.15-5.25
Did not wear gloves	1.06	0.12‒9.40	0.23	0.18-0.30	1.65	0.29-9.27
Used personal accessories	1.60	0.77-1.30	2.21	1.15-4.25	1.27	0.68-2.38
Used makeup	1.60	0.66-3.87	3.33	1.12-9.88	0.87	0.37-2.06
Had long nails	0.50	0.11-2.25	1.63	0.52-5.04	1.59	0.61-4.15
Did not disinfect the hands	1.29	0.62-2.67	0.45	0.24-0.84	0.86	0.46-1.61
Did not use hand disinfectant	3.90	1.42-11.12	0.31	0.11-0.82	1.70	0.60-4.79
Did not use paper towels to dry their hands after disinfection	0.15	0.15-0.20	0.74	0.68-0.80	0.76	0.70-0.82
Did not use a clean towel for drying hands after hand disinfection	3.14	1.22-8.06	0.36	0.15-0.85	1.73	0.69-4.33
Did not wash their hands	1.30	0.63-2.70	0.26	0.13-0.51	2.74	1.42-5.28
Not using soap to wash hands	1.32	0.62-2.82	0.39	0.20-0.77	0.97	0.51-1.82
Did not use clean water for hand washing	1.15	0.54-2.46	0.35	0.17-0.72	1.44	0.74-2.80
Did not use a clean towel to dry hands after washing them	1.27	0.60-2.71	0.57	0.30-1.05	1.98	1.05-3.72
Sanitary conditions outside the stall						
Garbage on the ground	0.92	0,39-2,17	9,66	0,47-1,95	0,83	0,39-1,76
Failure to use adequate trash cans	0.62	0,22-1,72	0,52	0,26-1,06	0,94	0,43-2,07
Presence of stagnant water at the stall	1.49	0,39-5,64	1,29	0,34-4,81	0,23	0,03-1,84
Stall had a non-washable floor	0.55	0,24-1,23	1,14	0,56-2,33	0,84	0,41-1,75
Presence of vectors at the stall	0.53	0,23-1,20	1,93	0,99-3,76	1,07	0,56-2,04
Presence of domestic animals at the stall	0.44	0,16-1,19	1,20	0,59-2,44	0,45	0,20-1,04
Sanitary conditions inside the sales stall						
Absence of drainage	0.83	0.39-1.73	0.73	0.40-1.35	0.82	0.44-1.53
Presence of garbage inside the stall	0.44	0.16-1.19	0.37	0.19-0.71	0.97	0.47-2.00
Failure to use adequate trash cans inside the stall	1.04	0.42-2.57	0.15	0.07-0.32	1.14	0.53-2.47
Presence of stagnant water inside the stall	1.81	0.35-9.39	0.73	0.67-0.79	1.08	0.21-5.55
Non-washable floor inside the stall	1.35	0.55-3.30	0.67	0.32-1.41	0.65	0.32-1.30
Presence of vectors inside the stall	0.72	0.31-1.69	1.64	0.80-3.37	1.50	0.77-2.92
Storage conditions of chicken meat						
Inadequate storage temperature	0.86	0.37-1.99	3.96	2.03-7.71	1.38	0.64-3.00
Contact of chicken meat with other meats at the counter	0.51	0.06-4.17	3.63	0.43-29.05	0.31	0.03-2.499

The following variables that were related to the presence of *E. coli* in chicken meat: the use of accessories by the handler (OR: 2.21; 95%CI: 1.15-4.25) and inadequate storage temperature (OR: 3.96; 95%CI 2.03-7.71) (Table 3).

The variables related to the presence of *S. aureus* in chicken meat were: the lack of apron use by the handler (OR: 2.46; 95%CI: 1.15-5.25), not washing the hands (OR: 2.74; 95%CI: 1.42-5.28) and not using a clean towel to dry the hands (OR: 1.98; 95%CI: 1.05-3.72).

## DISCUSSION

This study identified the association between the microbiological contamination of chicken meat in markets and some hygienic-sanitary conditions of the stalls and food handlers.

The presence of *Salmonella* spp. is associated with the non-use of hand sanitizers and not drying the hands after washing them. The main source of contamination of *Salmonella* spp. is human or animal feces ^5^^,^^25^. Hands can be contaminated by touching contaminated surfaces and handling meat contaminated with animal feces. It is likely that the presence of *Salmonella* spp. was due to the fact that the chicken meat was handled by people with contaminated hands and who did not use hand sanitizer, which would allow the microorganisms on the hands to be transmitted to the meat being marketed. The proper use of alcohol-based sanitizers decreases the quantity of microorganisms on the hands ^26^^,^^27^. Contamination by *Salmonella* spp. may also occur during poultry processing. Food of animal origin, such as poultry meat, can be contaminated with feces during processing ^13^. Flaws in this process facilitate contamination of the meat with pathogenic microorganisms, and thus, meat contaminated during the production process could transmit pathogens to marketing places, such as municipal markets. Therefore, if the handler comes into contact with meat contaminated with pathogenic microorganisms and subsequently handles non-contaminated meat, he/she can transfer these microorganisms to the non-contaminated food, resulting in cross-contamination ^1^.

Our results show that the presence of *E. coli* was associated with the use of personal accessories and the inadequate storage temperature of chicken meat. According to the good food handling practices, accessories used by handlers can be a source of contamination and inadequate food storage temperatures can contribute to microbial growth ^1^. Shiga toxin-producing *E. coli* can grow at temperatures ranging from 7°C to 50°C, with an optimum temperature of 37°C ^7^.

We identified an association between the use of aprons and the presence of *S. aureus*; however, no scientific evidence has been found to support that the use of aprons is an effective barrier to prevent food contamination by *S. aureus*. Not washing the hands and not drying them were also found to be associated with the presence of *S. aureus*. The skin and nostrils should be considered among the main sources of *S. aureus* infection ^28^. Failure to properly wash the hands and not drying them may increase the likelihood of chicken meat being contaminated with *S. aureus*^25^^,^^27^.

The prevalence rate of *Salmonella* spp. in our study (16%) was lower than the rate (56%) reported in fresh chicken meat from supermarkets during 2015 ^16^. The high prevalence of *E. coli* and *S. aureus* could explain why fewer samples were found to be infected with *Salmonella* spp. The presence of other bacteria may decrease the population of *Salmonella* spp. due to the low capacity of this bacterium to compete with other accompanying microorganisms ^29^.

The prevalence of *Salmonella* spp. found by our study is similar to that reported by previous research. For example, a study from Honduras reported a prevalence of 15% ^30^, which is similar to our results.

In our study, the prevalence of *E. coli* in chicken meat was 74%, which is higher than the 42% previously reported in Peru ^31^^)^ and also higher than what was reported in supermarkets in El Salvador during 2015 ^16^. It is likely that the high prevalence of *E. coli* was due to the deficient storage conditions of fresh chicken meat, and inadequate hygiene of the handler and the stalls^32^. The presence of *E. coli* in food suggests direct or indirect fecal contamination; this can occur during the processing of poultry ^1^^,^^13^, which is another possible source of this microorganism.

Most samples positive for *E. coli* exceeded 100 CFU/g, surpassing the acceptable the range ^23^. This may represent a greater health risk, since large quantities of microorganisms increase the probability of developing the disease ^11^. Several strains can be found in humans, which, in addition, have different degrees of virulence. Enteropathogenic *E. coli* is one of the main causes of diarrhea in developing countries and is potentially fatal in infants ^33^.

The presence of *S. aureus* indicates microbiological contamination by human handling. The prevalence of *S. aureus* in chicken meat was 24%, this exceeds what was found by a study in supermarkets during 2015 in two municipalities of El Salvador, which reported a prevalence of 13% in meat samples ^16^. *S. aureus* was mostly found to be within acceptable ranges, below 100 CFU/g. The presence of *S. aureus* represents a relative health risk, since it is part of the human microbiota, and in order to produce toxins and cause food poisoning it is necessary for colonies to be >10^5^ CFU/g ^34^. The quantity of microorganisms obtained during our study was not capable of producing toxins. Infections by *S. aureus* are particularly important in vulnerable populations ^33^. Some studies and laboratory tests have described resistance to some antibiotics ^35^.

One of the main limitations of the study is that we only selected markets from the departmental capitals because they have greater commercial activity and are located in settlements with large populations. The general conditions related to the marketing of chicken meat could be different in areas different from the departmental capitals. This study presents an approximation of the chicken meat contamination situation in El Salvador.

It was not possible to obtain samples from every stall in some of the markets, which limited data collection. This was due to the fact that it took between 3 to 4 hours to get to some of the municipal markets, therefore sampling started between 8:00am and 9:00am. However, the vendors arrive to the markets much earlier (between 5:00 am and 7:00 am) and finish selling their products before 9:00am. Therefore, the obtained sample (221 stalls) did not reach the calculated sample size (222 stalls). The difference was only 1 stall, which could influence the size of the confidence intervals used to infer our results about the population; however, the loss was minimal and there were no problems of statistical significance in our results. Another aspect that caused the loss of data was that many stalls that were censused at the beginning of the study planning changed their line of business (products sold) at the time of sampling. On the other hand, this was a crude association analysis, so some variables could lose significance in an adjusted model.

Since El Salvador does not have a regulation regarding microbiological parameters for chicken meat being sold at markets, our results had to be interpreted based on international regulations, which could contain aspects that are different from the reality of the country and that cannot always be adaptable. Internationally, each country should develop their own standards according to their conditions.

The hygienic-sanitary conditions of handlers and stalls are among the main factors associated with microbiological contamination in chicken meat marketed in El Salvador. This can lead to an increase in the prevalence of pathogenic microorganisms in chicken meat and contribute to an increase in food-related diseases. Therefore, appropriate regulations and improved food handling processes could contribute to improve this situation.

It is important to carry out interventions to improve the hygienic and sanitary conditions of the stalls and to train handlers in good handling and hygiene practices, in order to guarantee the safety and quality of chicken meat. Finally, it is necessary to carry out studies with methodological designs that allow the identification of factors directly related to the microbiological contamination of chicken meat.
