# Supplementary material for: Factors associated with microbiological contamination of chicken meat marketed in El Salvador
Source: Rev Peru Med Exp Salud Publica. 2023 Mar 27;40(1):25–33. doi: 10.17843/rpmesp.2023.401.12100 (PMC10953630; doi:10.17843/rpmesp.2023.401.12100)
Supplement: Translation

# Translation: Factores asociados a la contaminación microbiológica de la carne de pollo comercializada en El Salvador

**Authors:** Alejandro López, Tatiana Burgos, Marcela Vanegas, Zaida Álvarez, Yudy Mendez, Edgar Quinteros

**Affiliations:** 1 Instituto Nacional de Salud de El Salvador, San Salvador, El Salvador.; 2 Facultad de Química y Farmacia, Universidad de El Salvador, San Salvador, El Salvador.

**Keywords:** Alimentos, Pollos, Salmonella, Escherichia coli, Staphylococcus aureus

## Abstract

**Objetivo.:**

Determinar los factores higiénico-sanitarios asociados a la contaminación microbiológica de la carne de pollo comercializada en los mercados municipales de El Salvador.

**Materiales y métodos.:**

Se realizó un estudio transversal analítico en los 33 mercados municipales de las cabeceras departamentales de El Salvador. La muestra se calculó a partir de 456 puestos de venta, obteniendo un total de 256 puestos. Por cada puesto se obtuvo una muestra de carne de pollo. El análisis microbiológico se realizó en el Laboratorio Nacional de Salud Pública. Se calcularon frecuencias, porcentajes, medidas de tendencia central y de asociación utilizando SPSS versión 21.

**Resultados.:**

En el 74% de las muestras se encontró *Escherichia coli*, en el 24%, *Staphylococcus aureus* y en el 16%, *Salmonela* spp. La presencia de *Salmonella* spp, estuvo asociada con el no uso de desinfectante para las manos y no utilizar toalla para secarse las manos. La presencia de *E. coli* estuvo asociada al uso de accesorios personales y la inadecuada temperatura de almacenamiento. Mientras que la presencia de *S. aureus*, estuvo asociada a la falta de lavado de manos, no utilizar toalla para secarse las manos y no utilizar delantal.

**Conclusión.:**

Las condiciones higiénico-sanitarias de los manipuladores y de los puestos de venta están asociadas a la contaminación microbiológica en la carne de pollo comercializada en El Salvador.

## INTRODUCCIÓN

La contaminación microbiológica de los alimentos representa un riesgo para la salud pública, debido a que puede producir enfermedades conocidas comúnmente como Enfermedades Transmitidas por Alimentos (ETA) ^1^. La diarrea es el síntoma agudo más frecuente de las ETA, sin embargo, estas enfermedades pueden tener consecuencias graves como la insuficiencia renal, insuficiencia hepática, trastornos cerebrales y neurales, artritis reactiva, cáncer y la muerte ^2^. Las fallas en los procesos de producción de alimentos, manipulación inadecuada e insalubridad, son los principales factores que influyen en la contaminación. Los niños, ancianos y mujeres embarazadas son el grupo más vulnerable a las ETA ^1^. De acuerdo a la Organización Mundial de la Salud (OMS), las ETA causan alrededor de 420 000 muertes a nivel mundial cada año y afectan a una de cada diez personas que consumen alimentos contaminados ^3^. Entre el 2016 y 2020, el Sistema de Vigilancia Epidemiológica de El Salvador (VIGEPES) ^4^ reportó 1735 casos de intoxicación por alimentos y 1 482 613 casos de diarreas y gastroenteritis.

La *Salmonella* spp.*, Escherichia coli* (*E. coli*) y *Staphylococcus aureus* (*S. aureus)*son parte del grupo de microorganismos que producen las ETA ^3^. La *Salmonella* spp. es una bacteria que habita normalmente en el tracto intestinal de muchos animales, especialmente aves, bovinos, porcinos, reptiles, entre otros ^5^. La *Salmonella* spp. produce la enfermedad conocida como salmonelosis ^1^^,^^6^. 

La *E. coli* es una bacteria que se puede encontrar en el tracto intestinal de los humanos y animales de sangre caliente ^7^. La identificación de esta bacteria en el agua o alimentos se puede asociar con la contaminación fecal. Algunas cepas patógenas son capaces de secretar toxinas como Shiga ^8^ y ocasionar graves enfermedades ^9^. Por otra parte, el *S. aureus,* es una bacteria que habita principalmente en la piel y las fosas nasales de los humanos ^6^, pero puede llegar a los alimentos y producir gastroenteritis y además, dependiendo de la cantidad de Unidades Formadores de Colonias (UFC), puede generar enterotoxinas termorresistentes ^10^.

Los alimentos de origen animal son los más comúnmente relacionados con las ETA ^11^ y son los de mayor consumo entre la población ^3^. Por ejemplo, la carne de pollo en sus diferentes presentaciones, es una importante fuente de proteína y de nutrientes de alta calidad ^12^. La principal fuente de contaminación microbiológica de la carne de pollo es la microbiota autóctona de las aves de corral. La contaminación puede ocurrir debido a malas prácticas de manufactura y fallas en las diferentes etapas de producción y manipulación ^13^.

En El Salvador, una de las fuentes más importantes de proteína animal es la carne de pollo, debido a la disponibilidad y bajo costo ^14^. Entre los años 2018 y 2019, El Salvador reportó una producción de más de 280 millones de kilos de carne de pollo ^15^. En el país, los principales puntos de comercialización de carne de pollo son los supermercados y mercados municipales. Un estudio realizado en el 2015 en 43 supermercados de la capital de El Salvador, identificó una prevalencia de *Salmonella* spp. de 56%, una prevalencia de *E. coli* de 14% y una prevalencia de *S. aureus* de 13% ^16^, sin embargo, no se registran estudios similares en los mercados municipales, los cuales son los principales puntos de comercialización de carne de pollo debido a la accesibilidad económica de sus productos ^17^. Teniendo en cuenta que las condiciones sanitarias de estos sitios son deficientes, lo cual podría contribuir a la alta prevalencia de estos microorganismos, el objetivo del presente estudio fue determinar cuáles son los factores higiénico-sanitarios asociados a la contaminación por *Salmonella* spp*., E. coli y S. aureus* en la carne de pollo comercializada en los mercados municipales de las cabeceras departamentales de El Salvador.

**MENSAJES CLAVE**

Motivación para realizar el estudio. La presencia de microorganismos patógenos en los alimentos es un riesgo para la salud pública. Además, existe poca información en El Salvador acerca de contaminación microbiológica en alimentos comercializados en mercados municipales.

Principales hallazgos. Tres especies de microorganismos fueron identificadas en la carne de pollo; su presencia estuvo asociada con algunas condiciones higiénico-sanitarias de los manipuladores y de los puestos de venta.

Implicancias. Identificar los factores asociados a la contaminación microbiológica en muestras de pollo puede contribuir a la creación de políticas públicas enfocadas a fortalecer medidas preventivas y a difundir buenas prácticas de manipulación de alimentos.

## MATERIALES Y MÉTODOS

Se realizó un estudio transversal analítico a partir de muestras de carne de pollo crudo comercializada en puestos de venta de los 33 mercados municipales de las cabeceras departamentales de El Salvador, de agosto a noviembre de 2017. 

Para determinar la cantidad de muestras de pollo a incluir en el estudio se calculó una muestra de los puestos de venta de carne de pollo crudo en los mercados de las 14 cabeceras departamentales de El Salvador. Se realizó un levantamiento cartográfico de los puestos de venta con el apoyo de los inspectores de saneamiento ambiental del Ministerio de Salud, identificando un total de 456 puestos. Se elaboró un croquis de cada mercado y se ubicó cada uno de los puestos de venta de carne de pollo, las zonas de acuerdo con el rubro de comercialización y los puntos de referencia internos y externos (Figura 1). A cada puesto se le asignó un número correlativo y se identificó el nombre del propietario o arrendatario y el número de puesto asignado por la administración del mercado.

**Figura 1:** Ubicacion de las cabeceras departamentales donde estan ubicados los mercados incluidos en el estudio.

El cálculo de la muestra de los puestos de venta se realizó con una fórmula para poblaciones finitas ^18^, con una varianza de p=0,56 y q=0,44, con una significancia estadística del 1,96 y un error estadístico permitido de 5%. Se obtuvo una muestra de 222 puestos. La selección de la muestra de los puestos de venta se realizó con el método aleatorio simple a través del programa Microsoft Excel 2016. Por cada puesto de venta se obtuvo una muestra de carne de pollo, cada muestra estaba conformada por dos piezas de pollo crudo que se encontraban a la venta en el mostrador; todas las piezas colectadas estaban compuestas por piel y carne.

La recolección de la muestra fue realizada por investigadores del Instituto Nacional de Salud (INS) y personal de saneamiento ambiental del Ministerio de Salud de El Salvador. El encargado de recolectar la muestra explicó la importancia del estudio y solicitó el consentimiento informado por escrito a cada responsable de los puestos de venta. Posteriormente, se llenó un acta de toma de muestra en la que se detallaba la hora y fecha de la recolección, número de puesto y nombre del mercado, código, tipo de muestra, temperatura al momento de la recolección, la identificación del encargado o propietario del puesto y del encargado de la recolección de la muestra. Luego se llenó una hoja de inspección del establecimiento en la que se recolecto información sobre el uso de mascarilla, desinfección de manos, uso de accesorios personales, maquillaje, uso de delantal y guantes, lesiones en manos y piel, desagüe en el puesto de venta, presencia de vectores en el exterior e interior del puesto, presencia de basura en el exterior e interior del puesto de venta, presencia de agua estancada en el exterior e interior del puesto de venta y presencia de animales domésticos. 

Antes de tomar la muestra se realizaron mediciones de temperatura utilizando un termómetro para alimentos (COMARK DT400) con un rango de medición de -20°C a 100°C, el termómetro fue desinfectado antes de cada medición. Cada pieza de pollo fue embalada individualmente en una bolsa plástica con cierre hermético tipo *Whirl Pak* y fue identificada con una etiqueta adhesiva que contenía el código de la muestra, el análisis de laboratorio requerido, la hora, fecha y lugar de muestreo. Durante la recolección y transporte de las muestras se utilizó la metodología establecida por el CODEX Alimentarius ^19^ y los lineamientos para la toma y envío de muestras del Laboratorio Nacional de Salud Pública (LNSP). Las muestras fueron almacenadas y trasladadas al LNSP en hieleras a una temperatura de 4°C. Por cada muestra se llenó la hoja de toma, envío y recepción de muestras del LNSP.

El análisis de las muestras se realizó en el LNSP. El aislamiento e identificación de *Salmonella* spp. se realizó mediante la técnica de PCR Screening Assurance GDS, método oficial AOAC 2009.03 ^20^. Las muestras que resultaron inicialmente positivas se confirmaron por estriado en agares selectivos-diferenciales. Se aisló una colonia con aspecto típico de *Salmonella* spp. de cada uno de los medios selectivos-diferenciales. Se realizó la identificación de las cepas aisladas a través de los sistemas bioquímicos API 20E y VITEK 2. Esta metodología solo permite determinar la presencia o ausencia en 25g de muestra. 

La cuantificación de *E. coli* se realizó en placas *Petrifilm* a través del método oficial AOAC 991.14 ^21^. Las placas inoculadas se incubaron a 35ºC ± 1ºC por 48 horas. Para la lectura de las placas se contaron las colonias de color azul o rojo azulado, asociadas a la formación de burbujas de gas. El número de colonias obtenidas se multiplicó por el factor de dilución correspondiente. Los resultados de *E. coli* se expresan en unidades formadoras de colonias por gramo (UFC/g). 

La cuantificación de *S. aureus* se realizó en placa Petrifilm a través del método oficial AOAC 2003.11^22^. Las placas inoculadas se incubaron a 35ºC ± 1ºC por 24 horas. Para la lectura de las placas se contaron las colonias de color rojo-violeta. Para la confirmación de las colonias sospechosas de *S. aureus*, se colocó en las placas con crecimiento característico un disco de Petrifilm Staph Express (prueba DNasa). Se incubó a 35ºC ± 1ºC por 3 horas. Luego de la incubación se contaron las colonias de color rojo-violeta con una zona rosa alrededor de la colonia. Los aislados confirmados (DNasa positivos) de las placas Petrifilm se identificaron mediante la prueba de coagulasa. El resultado positivo indicó la presencia de *S. aureus*. El número de colonias confirmadas se multiplicó por el factor de dilución utilizado. Los resultados de *S. aureus* se expresan como UFC/g. 

Se creó una base de datos en Epi Info versión 7 con la información recolectada en la hoja de inspección del establecimiento, la hoja de inspección de las buenas prácticas de manipulación de alimentos y los resultados obtenidos del análisis de laboratorio. Posteriormente la base de datos fue exportada a *Statistical Package for the Social Sciences* (SPSS por sus siglas en inglés) versión 21, para realizar análisis de frecuencias, porcentaje de muestras positivas y medidas de tendencia central. Para el análisis de asociación se utilizó la prueba de razón de momios (OR) tomando como intervalo de confianza (IC) el 95%.

El estudio fue aprobado por el Comité Nacional de Ética de la Investigación en Salud, según acta de aprobación N° 19/2017, con el fin de dar cumplimiento a los principios de ética en investigación en salud. Todas las personas, propietarias de los puestos de venta aceptaron participar en el estudio y firmaron un consentimiento informado. Este documento especificaba los datos generales de los investigadores, la institución responsable, el objetivo del estudio, los procedimientos de selección y toma de muestra. Además, se hacía constar que era una participación totalmente voluntaria y confidencial. También se explicó a cada participante que las muestras podían ser vendidas o donadas.

## RESULTADOS

Se inspeccionaron las condiciones higiénico sanitarias de un total de 221 puestos de venta de carne de pollo cruda. La mayoría de los puestos de venta pertenecían a la cabecera departamental de San Salvador (24%), seguido de San Vicente (9,5%), Santa Ana (9,5%), Sonsonate (8,5%) y en menor proporción La Unión con 2,3%. El 75% de los puestos de venta contaba con desagüe, el 42% tenía presencia de vectores en el interior y el 32% en el exterior. En el 29% de los puestos se observó basura en el interior y en el 27% en el exterior. En el 29% de los puestos de venta se observó presencia de animales domésticos en el exterior (Figura 2). La temperatura promedio en la que se encontró la carne de pollo fue de 11,3 °C (DE: 8,6 °C). El 76,5% de las muestras se encontraron en una temperatura inadecuada (> 4°C) de almacenamiento.

En relación a las condiciones higiénico sanitarias de los manipuladores, el 97,3% no utilizaba guantes, el 83,7% no utilizaba delantal y el 48,9% se desinfectaban las manos. La mayoría de los manipuladores no presentó ningún tipo de lesión en las manos o en la piel (Tabla 1).

**Figura 2:** Principales condiciones sanitarias al interior y exterior de los puestos de venta de carne de pollo.

**Tabla 1:** Principales condiciones higiénicas de los manipuladores de carne de pollo (N=221).

Condiciones higiénicas del manipulador	Sí	No
	n (%)	n (%)
Uso de redecilla	51 (23,1)	170 (76,9)
Uso de delantal	36 (16,3)	185 (83,7)
Uso de guantes	6 (2,7)	215 (97,3)
Uso de accesorios personales	92 (41,6)	129 (58,4)
Utilizar maquillaje	37 (16,7)	184 (83,3)
Utilizar las uñas largas	22 (10,0)	199 (90,0)
Desinfección de las manos	108 (48,9)	113 (51,1)
Uso de desinfectante de mano	18 (8,1)	203 (91,9)
Uso de papel toallas para secar las manos después de realizar la desinfección	2 (0,9)	219 (99,1)
Uso de una toalla limpia para secarse las manos después de la desinfección de manos	24 (10,9)	197 (89,1)
Realizar el lavado de manos	107 (48,6)	113 (51,4)
Uso de jabón para lavarse las manos	133 (60,2)	88 (39,8)
Uso de agua limpia para lavarse las manos	139 (62,9)	82 (37,1)
Uso de una toalla limpia para secarse las manos después de haberlas lavado	82 (37,4)	137 (62,6)

El 16% de las muestras fueron positivas a *Salmonella* spp. Este resultado se encuentra fuera del estándar establecido por el Reglamento Técnico Centroamericano RTCA 67.04.50:17 ^23^. El 74% de las muestras presentó contaminación por *E. coli*, de las cuales, el 43 % se encontró en un rango aceptable y el 57% superó el estándar establecido, según el RTCA 67.04.50:17 ^23^ (Tabla 2). El 24% de las muestras presentó contaminación por *S. aureus*, de las cuales el 85% se encontró en un rango aceptable y el 15% rebasaba el estándar establecido según el estándar establecido en la Norma Nicaragüense NTON 03-023-12 ^24^ (Tabla 2).

**Tabla 2:** Presencia de *Salmonella* spp., *E. coli*, y *S. aureus* en carne de pollo comparado con criterios microbiológicos internacionales.

Microorganismo	N° de muestras positivas	Límites establecidos	N° de muestras por rango	Porcentaje (%)	Criterios microbiológicos
*Salmonella* spp.	35	Presencia	35	16	Ausencia en 25g^a^
		Ausencia	186	84	
*Escherichia coli*	164	<100 UFC/g	96	43	(m) 1x10^2^ UFC/g^a^
		>100 UFC/g	125	57	
*Staphylococcus aureus*	52	<100 UFC/g	188	85	(m) 1x10^2^ UFC/g^b^
		>500 UFC/g	33	15	

a Reglamento Técnico Centroamericano RTCA 67.04.50:1722

b Norma Nicaragüense NTON 03-023-1223

(m): Criterio microbiológico por debajo del cual el alimento no representa un riesgo para la salud

En la Tabla 3 se muestran las variables relacionadas a la contaminación microbiológica de la carne de pollo. La presencia de *Salmonella spp.* estuvo fuertemente asociada a no utilizar desinfectante para manos (OR: 3,97; IC95%: 1,42-11,12). De igual forma, no utilizar una toalla limpia para secarse las manos después de haberse desinfectado las manos, también se asoció con la presencia de *Salmonella* spp., (OR: 3,14; IC95%: 1,22-8,06).

**Tabla 3:** Variables relacionadas con la presencia de *Salmonella* spp., *E. coli* y *S. aureus* en la carne de pollo fresca.

Variable	***Salmonella* spp.**		*E. coli*		*S. aureus*	
	OR	IC95%	OR	IC95%	OR	IC95%
Condiciones higiénicas del manipulador						
No utilizar redecilla	1,67	0,75‒3,70	0,43	0,22-0,84	1,00	0,47-2,09
No utilizar delantal	0,62	0,20‒1,88	0,47	0,22-1,00	2,46	1,15-5,25
No utilizar guantes	1,06	0,12‒9,40	0,23	0,18-0,30	1,65	0,29-9,27
Utilizar accesorios personales	1,60	0,77-1,30	2,21	1,15-4,25	1,27	0,68-2,38
Utilizar maquillaje	1,60	0,66-3,87	3,33	1,12-9,88	0,87	0,37-2,06
Utilizar las uñas largas	0,50	0,11-2,25	1,63	0,52-5,04	1,59	0,61-4,15
No desinfectarse las manos	1,29	0,62-2,67	0,45	0,24-0,84	0,86	0,46-1,61
No utilizar desinfectante de mano	3,90	1,42-11,12	0,31	0,11-0,82	1,70	0,60-4,79
No utilizar papel toallas para secar las manos después de realizar la desinfección	0,15	0,15-0,20	0,74	0,68-0,80	0,76	0,70-0,82
No utilizar una toalla limpia para secarse las manos después de la desinfección de manos	3,14	1,22-8,06	0,36	0,15-0,85	1,73	0,69-4,33
No lavarse las manos	1,30	0,63-2,70	0,26	0,13-0,51	2,74	1,42-5,28
No utilizar jabón para lavarse las manos	1,32	0,62-2,82	0,39	0,20-0,77	0,97	0,51-1,82
No utilizar agua limpia para lavarse las manos	1,15	0,54-2,46	0,35	0,17-0,72	1,44	0,74-2,80
No utilizar una toalla limpia para secarse las manos después de haberlas lavado	1,27	0,60-2,71	0,57	0,30-1,05	1,98	1,05-3,72
Condiciones sanitarias en el exterior del sitio de venta						
Presencia de basura en el suelo	0,92	0,39-2,17	9,66	0,47-1,95	0,83	0,39-1,76
No utilizar basureros adecuados	0,62	0,22-1,72	0,52	0,26-1,06	0,94	0,43-2,07
Presencia de agua estancada en el sitio de venta	1,49	0,39-5,64	1,29	0,34-4,81	0,23	0,03-1,84
Piso no lavable en el sitio de venta	0,55	0,24-1,23	1,14	0,56-2,33	0,84	0,41-1,75
Presencia de vectores en el sitio de venta	0,53	0,23-1,20	1,93	0,99-3,76	1,07	0,56-2,04
Presencia de animales domésticos en el sitio de venta	0,44	0,16-1,19	1,20	0,59-2,44	0,45	0,20-1,04
Condiciones sanitarias en el interior del sitio de venta						
Ausencia de desagüe	0,83	0,39-1,73	0,73	0,40-1,35	0,82	0,44-1,53
Presencia de basura en el interior del sitio de venta	0,44	0,16-1,19	0,37	0,19-0,71	0,97	0,47-2,00
No utilizar basureros adecuados en el interior del sitio de venta	1,04	0,42-2,57	0,15	0,07-0,32	1,14	0,53-2,47
Presencia de agua estancada en el interior de la vivienda	1,81	0,35-9,39	0,73	0,67-0,79	1,08	0,21-5,55
Piso no lavable al interior del sitio de venta	1,35	0,55-3,30	0,67	0,32-1,41	0,65	0,32-1,30
Presencia de vectores en el interior del sitio de venta	0,72	0,31-1,69	1,64	0,80-3,37	1,50	0,77-2,92
Condiciones de almacenamiento de la carne de pollo						
Temperatura inadecuada de almacenamiento	0,86	0,37-1,99	3,96	2,03-7,71	1,38	0,64-3,00
Contacto de la carne de pollo con otras carnes en el mostrador	0,51	0,06-4,17	3,63	0,43-29,05	0,31	0,03-2,499

Las siguientes variables que estuvieron relacionadas con la presencia de *E. coli* en la carne de pollo fueron el uso de accesorios por parte del manipulador (OR: 2,21; IC95%: 1,15-4,25) y la temperatura inadecuada de almacenamiento (OR: 3,96; IC95 % 2,03-7,71) (Tabla 3).

Las variables relacionadas a la presencia de *S. aureus* en la carne de pollo fueron: la falta de uso de delantal por parte del manipulador (OR: 2,46; IC95%: 1,15-5,25), no lavarse las manos (OR: 2,74; IC95%: 1,42-5,28) y no utilizar una toalla limpia para secarse las manos (OR: 1,98; IC95%: 1,05-3,72). 

## DISCUSIÓN

Este estudio determinó la asociación entre la presencia de contaminación microbiológica en carne de pollo comercializada en mercados y algunas condiciones higiénico-sanitarias de los puestos de venta y de aquellos que manipulan los alimentos. 

La presencia de S*almonella* spp., está asociada con el no uso de desinfectantes de manos y no secarse las manos después de la desinfección. La *Salmonella spp.,* tiene como principal fuente de contaminación las heces fecales de humanos o animales ^5^^,^^25^. Las manos pueden contaminarse al tocar superficies contaminadas y al manipular carne contaminada con heces de animales. Es probable que la presencia de S*almonella* spp., se deba a que la carne de pollo fue manipulada por personas con las manos contaminadas y que no utilizaron desinfectante de mano, lo cual permitiría que los microorganismos de las manos fueran trasmitidos a la carne que se estaba comercializando. La adecuada utilización de desinfectantes a base de alcohol disminuye la cantidad de microorganismos presentes en las manos ^26^^,^^27^. Es posible que durante el proceso de faenado de las aves también ocurra contaminación por *Salmonella* spp. Los alimentos de origen animal, como la carne de aves, se pueden contaminar con heces fecales durante el proceso de faenado ^13^. Las fallas durante este proceso permiten que se contamine la carne con microorganismos patógenos, y de esta manera, la carne contaminada en el proceso de producción podría trasmitir patógenos a los puntos de comercialización, como los mercados municipales. Por lo tanto, si el manipulador entra en contacto con carne contaminada con microorganismos patógenos y posteriormente manipula carne no contaminada, puede transferir estos microorganismos al alimento no contaminado, generando contaminación cruzada ^1^. 

Nuestros resultados muestran que la presencia de *E. coli* estuvo asociada al uso de accesorios personales y a la temperatura inadecuada de almacenamiento de carne de pollo. Según las buenas prácticas de manipulación de alimentos, los accesorios utilizados por los manipuladores pueden ser fuente de contaminación y las temperaturas inadecuadas de almacenamiento de los alimentos pueden propiciar el crecimiento microbiano ^1^. La *E. coli* productora de toxina Shiga puede crecer a temperaturas que oscilan entre 7°C y 50°C, con una temperatura óptima de 37ºC ^(^^7^.

Identificamos una asociación entre el uso de delantal y la presencia de *S. aureus*, sin embargo, no se ha encontrado evidencia científica que demuestre que el uso de delantal represente una barrera efectiva para evitar la contaminación de los alimentos por *S. aureus*. También se identificó que no practicar el lavado de manos y no secarse las manos también estaban asociados a la presencia de *S. aureus*. Se debe considerar que la piel y las fosas nasales son unas de las principales fuentes de infección por *S. aureus*^28^. No realizar un correcto lavado de manos y posterior secado puede aumentar la probabilidad de que la carne de pollo sea contaminada por *S. aureus*^25^^,^^27^. 

La prevalencia de *Salmonella* spp., es menor a la reportada en supermercados en 2015, año durante el cual se identificó una prevalencia del 56% en carne fresca de pollo ^16^, en contraste al 16% encontrado en el presente estudio. Una mayor presencia de *E. coli* y *S. aureus,* podría explicar por qué se identificó menos muestras con *Salmonella* spp. La presencia elevada de otras bacterias pueden disminuir la población de *Salmonella* spp., debido a la poca capacidad de esta bacteria para competir con otros microorganismos acompañantes ^29^. 

La prevalencia de *Salmonella* spp. encontrada en nuestro estudio fue similar a la reportada por investigaciones previas. Por ejemplo, un estudio llevado a cabo en Honduras reportó una prevalencia del 15% ^30^, similar a la reportada en el presente estudio. 

La prevalencia de *E. coli* en carne de pollo fue del 74%, un porcentaje mayor al 42% reportado previamente en Perú ^31^ y mayor también que la prevalencia reportada en supermercados de El Salvador en el año 2015 ^16^. Es probable que el porcentaje elevado de *E. coli*, se deba a las condiciones de almacenamiento de la carne fresca de pollo, y a la deficiencia en los principios generales de higiene del manipulador y los puestos de venta ^32^. La presencia *de E. coli* en un alimento indica una contaminación directa o indirecta de origen fecal; esto puede ocurrir en el proceso del sacrificio o el faenado de las aves ^1^^,^^13^, la cual es otra posible fuente de origen de este microorganismo. 

La mayoría de muestras con presencia de *E. coli* presentó valores por encima de los rangos aceptables, superando las 100 UFC/g ^23^. Esto puede representar un mayor riesgo para la salud, debido a que las grandes cantidades de microorganismos aumentan la probabilidad de ocasionar alguna enfermedad ^11^. Numerosas cepas se pueden encontrar en enfermedades humanas, las cuales, además, presentan diferentes grados de virulencia. La *E. coli* entero patógena es una de las principales causantes de diarrea en los países en desarrollo y es potencialmente mortal en lactantes ^33^. 

La presencia de *S. aureus* es un indicador de contaminación microbiológica por manipulación humana. La prevalencia encontrada en la carne de pollo fue del 24%, lo cual supera los valores encontrados por un estudio realizado en supermercados durante el año 2015 en dos municipios del El Salvador, el cual reportó una prevalencia del 13% en las muestras de carne recolectadas ^16^. El *S. aureus* fue cuantificado en su mayoría dentro de rangos aceptables, por debajo de 100 UFC/g. La presencia de *S. aureus* representa un riesgo relativo a la salud, debido que forma parte de la microbiota en humanos y para ocasionar una intoxicación alimentaria es necesario que las colonias sean >10^5^ UFC/g para producir toxinas ^34^. Los resultados obtenidos en este estudio determinan que, la cantidad recuperada de este microorganismo no es capaz de producir toxinas. Las infecciones ocasionadas por *S. aureus* pueden ser de importancia en poblaciones vulnerables ^33^. Algunos estudios y pruebas de laboratorio han descrito resistencia a algunos antibióticos ^35^.

Dentro de las principales limitaciones del estudio se puede mencionar que solo se seleccionaron aquellos mercados pertenecientes a las cabeceras departamentales por tener mayor actividad económica y asentamientos poblacionales de mayor tamaño. Las condiciones en general, relacionadas a la comercialización de la carne de pollo, podrían ser diferentes en zonas que no pertenecen a las cabeceras departamentales. Este estudio, presenta una aproximación a la situación de la contaminación en la carne de pollo en El Salvador.

En algunos mercados no se logró tomar muestras de carne de pollo de todos los puestos seleccionados, lo cual limitó la recolección de datos. Esto ocurrió debido a que el traslado hacia algunos mercados municipales demandó entre 3 a 4 horas, llegando al lugar de muestreo entre las 8:00 am y 9:00 am. Sin embargo, los comerciantes terminan sus ventas antes de esa hora y asisten a los mercados horas antes (entre las 5:00 am a 7:00 am) para comercializar sus productos. Por lo tanto, la muestra obtenida (221 puestos) no alcanzó el tamaño original de la muestra (222 puestos). La diferencia fue únicamente 1 puesto de venta, lo cual podría influir en los tamaños de los intervalos de confianza utilizados para hacer inferencia de nuestros resultados sobre la población, sin embargo, la perdida fue mínima, por lo tanto, no se presentaron problemas de significancia estadística en los resultados. Otro aspecto que ocasionó la pérdida de datos fue que muchos puestos que fueron censados al inicio de la planificación del estudio, cambiaron de giro (productos que comercializan) al momento de realizar la toma de muestra. Por otro lado, el análisis realizado corresponde a un análisis de asociación crudo, por lo que algunas variables podrían perder significancia en un modelo ajustado.

Debido a que en El Salvador no se cuenta con una reglamentación que establezca parámetros microbiológicos en la carne de pollo en puntos de comercialización, la interpretación de los resultados tuvo que basarse en reglamentación internacional, la cual podría contener aspectos diferentes a la realidad del país y que no siempre pueden ser adaptables. Internacionalmente se recomienda que cada país elabore sus propios estándares de acuerdo a sus condiciones. 

Las condiciones higiénico-sanitarias de los manipuladores y de los puestos de venta se encuentran entre los principales factores asociados a la contaminación microbiológica en la carne de pollo comercializada en El Salvador. Esto puede generar un aumento en la prevalencia de microorganismos patógenos en la carne de pollo y contribuir con el aumento de enfermedades relacionadas con los alimentos. Por lo tanto, una normativa apropiada y la mejora de los procesos de manipulación de alimentos podrían contribuir a mejorar esta situación.

Es importante realizar intervenciones para mejorar las condiciones higiénico sanitarias de los puestos de venta y capacitar a los manipuladores sobre las buenas prácticas de manipulación e higiene que garanticen la inocuidad y la calidad de la carne de pollo. Finalmente, es necesario ejecutar estudios con diseños metodológicos que permitan identificar los factores directamente relacionados con la contaminación microbiológica de la carne de pollo.
